# Immune Organoids: A Review of Their Applications in Cancer and Autoimmune Disease Immunotherapy

**DOI:** 10.3390/cimb47080653

**Published:** 2025-08-13

**Authors:** David B. Olawade, Emmanuel O. Oisakede, Eghosasere Egbon, Saak V. Ovsepian, Stergios Boussios

**Affiliations:** 1 Department of Allied and Public Health, School of Health, Sport and Bioscience, University of East London, London E16 2RD, UK; d.olawade@uel.ac.uk; 2 Department of Research and Innovation, Medway NHS Foundation Trust, Gillingham Kent ME7 5NY, UK; 3 Department of Public Health, York St John University, London E14 2BA, UK; 4 Department of Clinical Oncology, Leeds Teaching Hospitals Trust, Leeds LS9 7LP, UK; emmanuel.oisakede@nhs.net; 5 Department of Health Research, University of Leeds, Leeds LS2 9JT, UK; 6 Department of Tissue Engineering and Regenerative Medicine, Faculty of Life Science Engineering, FH Technikum, 1200 Vienna, Austria; eghosaseregabriel@gmail.com; 7 Faculty of Engineering and Science, University of Greenwich London, Chatham Maritime Kent ME4 4TB, UK; s.v.ovsepian@greenwich.ac.uk; 8 Faculty of Medicine, Tbilisi State University, Tbilisi 0177, Georgia; 9 Faculty of Medicine, School of Health Sciences, University of Ioannina, 45110 Ioannina, Greece; 10 Department of Medical Oncology, Ioannina University Hospital, 45500 Ioannina, Greece; 11 Faculty of Medicine, Health and Social Care, Canterbury Christ Church University, Canterbury CT1 1QU, UK; 12 Faculty of Life Sciences & Medicine, School of Cancer & Pharmaceutical Sciences, King’s College London, Strand, London WC2R 2LS, UK; 13 AELIA Organization, 9th Km Thessaloniki—Thermi, 57001 Thessaloniki, Greece

**Keywords:** immune organoids, cancer immunotherapy, autoimmune diseases, precision medicine, bioengineering

## Abstract

Immune organoids have emerged as a ground-breaking platform in immunology, offering a physiologically relevant and controllable environment to model human immune responses and evaluate immunotherapeutic strategies. Derived from stem cells or primary tissues, these three-dimensional constructs recapitulate key aspects of lymphoid tissue architecture, cellular diversity, and functional dynamics, providing a more accurate alternative to traditional two-dimensional cultures and animal models. Their ability to mimic complex immune microenvironments has positioned immune organoids at the forefront of cancer immunotherapy development, autoimmune disease modeling, and personalized medicine. This narrative review highlights the advances in immune organoid technology, with a focus on their applications in testing immunotherapies, such as checkpoint inhibitors, CAR-T cells, and cancer vaccines. It also explores how immune organoids facilitate the study of autoimmune disease pathogenesis with insights into their molecular basis and support in high-throughput drug screening. Despite their transformative potential, immune organoids face significant challenges, including the replication of systemic immune interactions, standardization of fabrication protocols, scalability limitations, biological heterogeneity, and the absence of vascularization, which restricts organoid size and maturation. Future directions emphasize the integration of immune organoids with multi-organ systems to better replicate systemic physiology, the development of advanced biomaterials that closely mimic lymphoid extracellular matrices, the incorporation of artificial intelligence (AI) to optimize organoid production and data analysis, and the rigorous clinical validation of organoid-derived findings. Continued innovation and interdisciplinary collaboration will be essential to overcome existing barriers, enabling the widespread adoption of immune organoids as indispensable tools for advancing immunotherapy, vaccine development, and precision medicine.

## 1. Introduction

The immune system is an intricate network of cells, tissues, and signaling molecules that collectively protect the body from infections, eliminate harmful agents, and maintain internal stability or homeostasis [1]. While traditional research models have contributed significantly to our understanding of immune function, they present critical limitations that immune organoids are uniquely positioned to address [2].

Traditional two-dimensional (2D) cell cultures, though offering simplicity and reproducibility, fundamentally lack the cellular interactions and three-dimensional architecture essential for replicating in vivo immune environments [3,4]. Animal models, despite their systemic complexity, often exhibit species-specific differences that limit direct translation to human immune responses [5]. These limitations create a critical gap in our ability to accurately model human immune pathogenesis and predict therapeutic responses.

Immune organoids bridge this translational gap by providing intermediate complexity that combines the experimental control of in vitro systems with the physiological relevance approaching in vivo conditions. These 3D structures are multicellular self-organized tissues that mimic the architecture, functions, and complexities of their corresponding in vivo organs [6,7]. Conceptually, immune organoids occupy a unique position in the research model continuum, offering higher physiological relevance than 2D cultures while maintaining greater experimental control than animal models [8,9,10].

In recent years, advances in bioengineering and stem cell biology have enabled the development of immune organoids that partially recreate the microenvironments of lymphoid tissues, such as lymph nodes, spleen, and thymus. These models facilitate the study of specific immune processes, including antigen presentation, T-cell activation, B-cell maturation, and cytokine production [11,12]. However, current immune organoid models typically replicate only selected features of immune tissues rather than their full cellular and structural complexity [13,14,15].

The transformative potential of immune organoids lies in their applications across cancer immunotherapy and autoimmune disease research. In oncology, they enable testing of checkpoint inhibitors [16] and CAR-T cells under human-relevant conditions [17]. For autoimmune diseases, they provide platforms to explore immune dysregulation mechanisms [18] and screen immunomodulatory therapies [19]. Additionally, they show promise in infectious disease research and vaccine development [20].

While immune organoids successfully address the physiological irrelevance of 2D cultures and the species-specificity limitations of animal models, critical challenges remain in replicating systemic immune interactions, achieving standardization of fabrication protocols, and scaling production for clinical applications. These limitations currently restrict their widespread adoption as predictive models for immunotherapy development. This narrative review provides a comprehensive analysis of immune organoid development and applications, with particular emphasis on cancer immunotherapy and autoimmune disease research. We examine current fabrication techniques, therapeutic applications, and the challenges limiting clinical translation, while proposing evidence-based solutions and future directions for this rapidly evolving field.

## 2. Methods

This narrative review was conducted to synthesize and critically appraise the existing literature on the development and applications of immune organoids in cancer immunotherapy and autoimmune disease research. The methodology followed a structured yet flexible approach consistent with accepted practices for narrative reviews, aiming to offer comprehensive insights into recent advancements, current challenges, and future directions in the field.

### 2.1. Search Strategy and Sources

An extensive literature search was conducted across multiple electronic databases, including PubMed, Scopus, Web of Science, Google Scholar, and ScienceDirect, covering publications from 2000 to April 2025. Search terms included combinations of keywords such as “immune organoids,” “cancer immunotherapy,” “autoimmune diseases,” “immune-oncology,” “organoid models,” “3D culture,” “CAR-T cells,” “checkpoint inhibitors,” and “immune system modeling.” Boolean operators (AND, OR) were used to expand or limit search queries. Priority was given to peer-reviewed original research articles, systematic reviews, and high-impact narrative reviews. Supplementary searches included the manual screening of reference lists from relevant articles and gray literature, including preprints and regulatory documents.

### 2.2. Inclusion and Exclusion Criteria

Studies were included if they:

- Focused on the design, fabrication, or application of immune organoids;
- Explored immune organoids in relation to cancer immunotherapy, autoimmune disease modeling, or drug testing;
- Were published in English and available in full text.

Exclusion criteria comprised:

- Studies limited solely to non-immune organoid systems (e.g., brain or liver organoids without immune components);
- Articles not related to human immune modeling (e.g., plant or microbial organoids);
- Editorials, opinion pieces, and news articles without substantive scientific data.

### 2.3. Data Extraction and Synthesis

Relevant information was extracted from selected studies, including study objectives, methodologies, organoid type (stem cell-derived, tissue-derived, or bioprinted), cell sources, immune cell components, applications in immunotherapy or disease modeling, reported outcomes, limitations, and scalability challenges. Data synthesis involved thematic analysis, whereby studies were grouped under conceptual themes, such as organoid development, immunotherapy applications, fabrication techniques, and technological limitations. Tables were generated to summarize key features of immune organoids, comparative advantages over traditional models, advances in co-culture systems, and integration with artificial intelligence (AI) and biomaterials.

### 2.4. Quality Appraisal

Although formal risk of bias tools is not standard in narrative reviews, efforts were made to prioritize high-quality studies from reputable journals. Where possible, findings were cross-referenced with existing systematic reviews or meta-analyses to assess the consistency. Recent developments and innovations were critically evaluated for maturity, reproducibility, and translational potential.

## 3. Development of Immune Organoids

The development of immune organoids has revolutionized the study of human immune function by providing a sophisticated platform to replicate selected aspects of the complex interactions within the immune system. Unlike traditional models, immune organoids enable researchers to recreate 3D structures with cellular diversity, structural organization, and functional features reflective of in vivo systems, though a full replication of organ-level complexity remains a future goal [21]. Table 1 compares immune organoids with traditional 2D cultures and animal models, emphasizing their superior physiological relevance and predictive value for human responses.

### 3.1. Definition and Characteristics

Immune organoids are 3D bioengineered constructs designed to mimic critical features of immune tissues [6,7]. They are derived from stem cells or primary tissues and emulate key structures, such as lymph nodes, spleen, and thymus. One defining feature is their cellular diversity, which includes immune cell types, like T cells, B cells, macrophages, dendritic cells, and stromal cells. This heterogeneity is essential for reproducing immune processes, such as antigen presentation, T-cell activation, and antibody production. For instance, B cells within these organoids can undergo class-switch recombination and somatic hypermutation, crucial steps for producing high-affinity antibodies [26].

**Figure 1:**
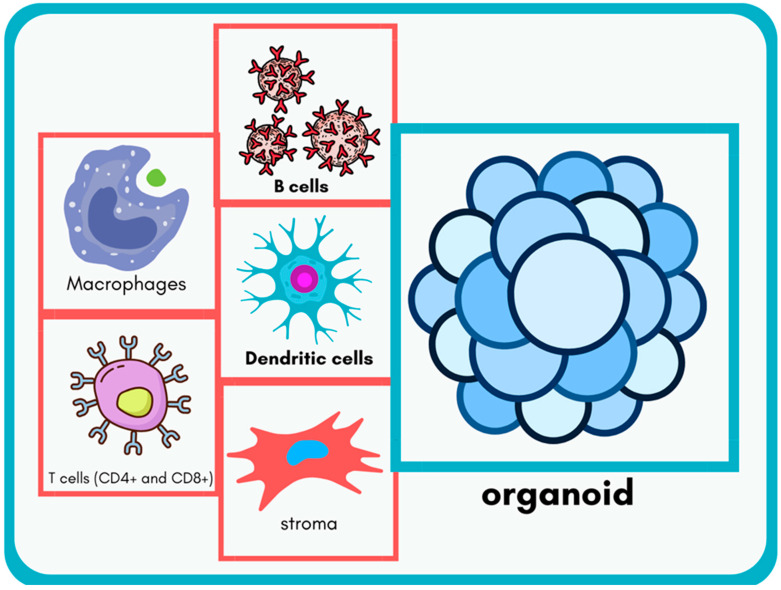
Schematic illustration of an immune organoid highlighting its key cellular components. The organoid is composed of diverse immune cell types, including macrophages, dendritic cells, B cells, T cells (CD4^+^ and CD8^+^), and stromal cells, reflecting the cellular heterogeneity and organization found in natural lymphoid tissues. This diversity is essential for modeling critical immune functions, such as antigen presentation, T-cell activation, and antibody production.

Another hallmark is structural organization, which facilitates cellular interactions in a spatially defined manner, akin to natural immune tissues. For example, lymphoid organoids can recreate follicular structures where B cells interact with T follicular helper cells, providing insights into germinal center dynamics [27]. Functionally, immune organoids simulate key immune processes, including cytokine secretion and antigen-specific T-cell proliferation. Nonetheless, it must be emphasized that current organoid models generally allow the exploration of a subset of immune functions rather than fully replicating immune tissue complexity. The defining features of immune organoids, including their cellular diversity and structural organization, are depicted in Figure 1, which illustrates the main immune cell types and stromal elements that collectively recapitulate the complexity of natural immune tissues. On a molecular level, immune organoids enable the study of intracellular signaling cascades central to immune activation. For instance, antigen presentation by dendritic cells and macrophages triggers T-cell receptor (TCR) signaling in CD4^+^ and CD8^+^ T cells via the ZAP-70–LAT–SLP76 axis, leading to NF-κB, AP-1, and NFAT activation [28,29]. B-cell maturation within organoids is governed by signals through the B-cell receptor (BCR) complex and co-stimulation via CD40-CD40L interactions, activating downstream pathways, such as PI3K/AKT and MAPK, to drive class-switch recombination and somatic hypermutation [30]. Furthermore, cytokine secretion (e.g., IL-2, IL-21, IFN-γ) and receptor engagement (e.g., IL-2Rα) trigger the JAK/STAT signaling axis, shaping T-cell expansion and fate decisions [31].

### 3.2. Fabrication Techniques

The fabrication of immune organoids involves sophisticated bioengineering techniques, each offering distinct advantages depending on research objectives. To guide method selection, we present each approach using a unified framework of advantages, limitations, and key applications.

#### 3.2.1. Stem Cell-Derived Models

The advantages of stem cell-derived models include their exceptional scalability, as iPSCs and ESCs provide unlimited cell sources for large-scale production, and their potential for standardization through consistent genetic backgrounds that reduce donor variability. These models offer superior controllability through defined differentiation protocols that enable precise lineage specification, and they excel in disease modeling by incorporating patient-specific mutations for personalized studies.

However, several limitations constrain their utility. The extended maturation time, requiring weeks to months for functional immune cell development, poses logistical challenges for time-sensitive applications. Additionally, these models may exhibit incomplete differentiation, lacking full phenotypic maturation compared to primary cells, and they require substantial technical complexity demanding specialized expertise in stem cell culture and differentiation protocols.

Stem cell-derived models rely on the pluripotent capacity of induced pluripotent stem cells (iPSCs) or embryonic stem cells (ESCs), which are directed to differentiate into immune cell lineages under tightly controlled conditions. Recent advances in differentiation protocols have enabled the generation of thymic organoids that support T-cell maturation, using defined combinations of growth factors and cytokines to mimic thymic stromal environments [32,33]. These models provide valuable insights into immune cell development and are especially useful for studying congenital immunodeficiencies and T-cell-based immunotherapies.

Molecular control over lineage specification is achieved using stage-specific exposure to morphogens and transcriptional regulators. For example, differentiation into thymic epithelial lineage involves the activation of FOXN1, a master regulator of thymic organogenesis, modulated by BMP4 and FGF7 signaling [34]. Similarly, Notch signaling via DLL4 and Notch1 engagement is critical for T-cell lineage commitment from hematopoietic progenitors, activating downstream genes, such as GATA3, BCL11B, and TCF7 [35].

#### 3.2.2. Tissue-Derived Models

Tissue-derived models offer significant advantages through their preservation of native complexity, maintaining endogenous cellular heterogeneity and tissue architecture that closely reflects physiological conditions. These models provide superior physiological relevance by preserving natural cell–cell interactions and signaling networks, enable rapid establishment with shorter timeframes to achieve functional organoids compared to stem cell approaches, and demonstrate excellent disease specificity by capturing patient-specific pathological features.

Nevertheless, these models face important limitations, including substantial donor variability that creates significant heterogeneity between different tissue sources, limited scalability due to dependence on primary tissue availability, standardization challenges that make it difficult to achieve consistent protocols across donors, and ethical considerations requiring fresh tissue procurement from patients.

Tissue-derived models use primary lymphoid tissues, such as the tonsils, lymph nodes, or spleen, to isolate and culture immune cells within a three-dimensional framework. This approach retains much of the native cellular heterogeneity and tissue architecture, allowing for more physiologically accurate modeling of immune responses. Tonsil-derived organoids, for example, have demonstrated the ability to recapitulate key immunological features, such as germinal center formation and antigen-driven antibody production, making them suitable for studying humoral immunity [4,36].

#### 3.2.3. Three-Dimensional Bioprinting Approaches

Three-dimensional bioprinting approaches provide unique advantages through their spatial precision, enabling the controlled positioning of different cell types and ECM components with unprecedented accuracy. These methods offer exceptional architectural control, allowing the recreation of complex tissue zonation and compartmentalization, provide extensive customization opportunities for designing organoids with specific structural features, and enhance reproducibility through standardized printing protocols that reduce batch-to-batch variation.

However, significant limitations constrain their widespread adoption. Technical barriers require specialized equipment and bioink formulation expertise that may not be readily available in all laboratories. The printing process itself may compromise cell viability and function, while the current resolution limits prevent the achievement of single-cell precision. Additionally, cost considerations include higher initial investment and operational expenses compared to conventional organoid culture methods.

Three-dimensional bioprinting represents a cutting-edge technique that allows the precise spatial arrangement of immune cells and extracellular matrix (ECM) components through the layer-by-layer deposition of bio-inks. This method has enabled the creation of organoid structures that mimic lymphoid tissue compartments, facilitating the study of immune cell migration, antigen presentation, and vaccine efficacy. Bioprinted models have already been used to evaluate vaccine candidates by reproducing functional zones akin to lymphoid follicles [26,37,38].

#### 3.2.4. Method Selection Decision Framework

To assist researchers in selecting the optimal fabrication approach, we propose a comprehensive decision framework based on specific research requirements and constraints. Stem cell-derived models should be chosen when large-scale, standardized production is required, when studying genetic diseases or rare immune disorders, when long-term scalability is a priority, or when standardization across multiple laboratories is essential for reproducible results.

Tissue-derived models represent the optimal choice when maximum physiological relevance is critical for the research question, when studying patient-specific responses or disease heterogeneity, when rapid organoid establishment is needed for time-sensitive applications, or when native immune architecture preservation is essential for accurate modeling.

**Figure 2:**
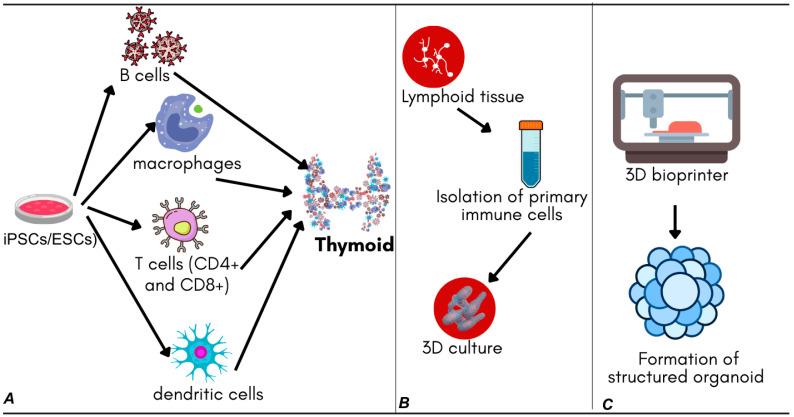
Overview of major fabrication techniques for immune organoids. (**A**) Stem cell-derived organoids: pluripotent stem cells (iPSCs/ESCs) are differentiated into immune cell lineages (B cells, macrophages, T cells, and dendritic cells) using defined growth factors and cytokines, leading to the formation of thymic organoids. (**B**) Tissue-derived organoids: primary immune cells are isolated from lymphoid tissues and cultured in 3D to preserve native cellular heterogeneity and architecture. (**C**) 3D bioprinting: immune cells and ECM components are deposited in a layer-by-layer manner using a 3D bioprinter, enabling precise spatial arrangement and formation of structured organoids.

Three-dimensional bioprinting approaches should be selected when precise spatial control over cell positioning is required for the experimental design, when studying immune cell migration or tissue zonation phenomena, when custom organoid architectures are needed for specific applications, or when standardized, reproducible protocols are a priority for multi-site studies. Figure 2 provides a visual overview of these fabrication strategies and their respective applications.

Despite these advancements, several challenges persist across all approaches, including achieving full functional maturation of immune cells, ensuring reproducibility between batches, and developing protocols that allow for large-scale production suitable for translational research and drug screening applications.

## 4. Applications for Immunotherapy Development

The development of immune organoids has opened transformative avenues in immunotherapy research, providing a human-relevant platform to study aspects of immune responses, develop novel therapies, and optimize existing treatments. Although immune organoids replicate important elements of the immune microenvironment, it is important to recognize that their current complexity allows the exploration of selected immune functions rather than full replication of in vivo immune tissues. In the context of cancer and autoimmune disease research, immune organoids, often in combination with tumor-derived organoids or co-cultures, have emerged as promising tools to enhance precision medicine efforts. Table 2 categorizes different types of immune organoids, such as lymphoid and thymic organoids, highlighting their source materials and specific applications.

### 4.1. Cancer Immunotherapy

Immune organoids offer new opportunities for studying tumor–immune interactions, albeit primarily through co-culture systems with tumor organoids rather than as stand-alone immune structures. Their ability to recreate specific immune microenvironments provides valuable insights into therapeutic efficacy and mechanism of action, although they currently model only a subset of the complex tumor–immune dynamics. Notably, they allow the interrogation of immune checkpoint blockade, CAR-T-cell activation, and, to a lesser extent, cancer vaccine responses. However, the translational utility of immune organoids depends on how well these models predict actual clinical outcomes.

Checkpoint Inhibitors, such as those targeting PD-1/PD-L1 and CTLA-4 pathways, have revolutionized cancer therapy. Co-culturing tumor-derived organoids with immune cells or immune organoids has enabled researchers to evaluate checkpoint inhibitor activity in a setting that approximates human physiology [44]. However, pure immune organoid models for studying checkpoint blockade without the presence of tumor cells remain largely undeveloped. Molecular interrogation within co-cultured immune organoids allows for the quantification of PD-1/PD-L1 pathway suppression of TCR-mediated signaling [45]. Engagement of PD-1 with PD-L1 inhibits PI3K and Ras-MAPK pathways through SHP2 recruitment, attenuating T-cell activation and cytokine secretion [46]. The restoration of IL-2, IFN-γ, and granzyme B expression following anti-PD-1 treatment serves as a readout for functional reactivation [47]. These represent robust proof-of-concept findings. However, only a few studies have correlated ex vivo immune organoid responses with clinical treatment outcomes. Dijkstra et al. [48] demonstrated that patient-derived tumor organoid co-cultures with autologous T cells could predict immune checkpoint blockade responsiveness, including resistance patterns that mirrored clinical non-response. While promising, these findings remain preliminary.

CAR-T-cell therapy similarly benefits from co-culture approaches. On a molecular level, CAR-T cells in immune organoids have shown intracellular calcium flux and the upregulation of effector genes (e.g., GZMB, IFNG, TNF) upon antigen recognition, reflecting real-time activation through synthetic CD3ζ and 4-1BB/CD28 domains [49]. Organoid systems permit the analysis of early transcriptional events using single-cell RNA-seq, capturing signatures of T-cell exhaustion or memory differentiation post-therapy [50]. Immune cells, including CAR-T cells, have been tested for cytotoxic activity against tumor organoids, providing a functional readout of efficacy, persistence, and off-target effects [51]. Despite these mechanistic insights, the current studies remain preclinical and have not yet demonstrated a predictive correlation with CAR-T efficacy or toxicity in patients.

**Figure 3:**
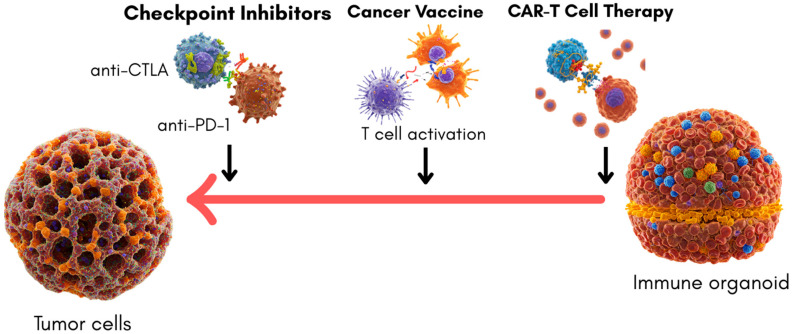
Schematic representation of immune organoid applications in cancer immunotherapy. Tumor organoids are co-cultured with immune organoids to model the effects of checkpoint inhibitors (anti-CTLA-4, anti-PD-1), cancer vaccines (T-cell activation), and CAR-T-cell therapy (targeted tumor cell killing). This approach enables the study of tumor–immune interactions and the evaluation of immunotherapeutic efficacy in a physiologically relevant 3D environment.

Cancer vaccines represent another promising area where immune organoid systems could eventually be impactful. Current models typically involve the use of tumor organoids or lymphoid-like structures in co-culture settings to simulate antigen presentation and T-cell activation [52,53,54,55]. For example, inspired by lymph node architecture, 3D lymphoid aggregates have been used to study dendritic cell maturation and vaccine-driven T-cell activation [56,57]. Yet, these remain early-stage platforms, with no validated correlation between in vitro vaccine responses and actual patient immunogenicity or tumor control. Thus, while conceptually promising, vaccine-related organoid systems remain in early exploratory phases. A summary of how immune organoids are used to model and evaluate major cancer immunotherapy strategies and also “clinical Readiness” scale to guide translational interpretation is illustrated in Figure 3 and Table 3, respectively.

Overall, while these 3D models represent a substantial improvement over traditional 2D assays, their current use in translational immunotherapy research is largely exploratory. Further progress will require harmonized protocols, larger patient cohorts, and longitudinal validation of organoid-derived biomarkers in prospective clinical trials.

### 4.2. Autoimmune Disease Research

Autoimmune diseases present unique research challenges due to their complex pathophysiology and significant patient-to-patient variability [58]. Immune organoids offer promising but still developing platforms for studying specific aspects of autoimmune dysregulation, with applications varying considerably across different disease contexts.

#### 4.2.1. Disease-Specific Organoid Applications

Rheumatoid Arthritis (RA) Models: RA organoid models primarily focus on synovial tissue–immune cell interactions, emphasizing the role of Th17/Treg imbalances in joint destruction. These models typically incorporate synovial fibroblasts, macrophages, and T-cell subsets to study inflammatory cascades involving TNF-α, IL-1β, and IL-17 [59]. Success rates for establishing functional RA organoids are in the range of 60–80% depending on tissue source and culture conditions. Key cellular components include elevated Th17:Treg ratios (typically 3:1 compared to 1:1 in healthy controls) and activated M1 macrophages expressing high levels of CD68 and TNF-α [43].

Systemic Lupus Erythematosus (SLE) Models: SLE organoids emphasize autoantibody production and B-cell dysregulation, often incorporating patient-derived B cells, plasma cells, and dendritic cells. These models focus on germinal center dysfunction and aberrant class-switch recombination leading to anti-nuclear antibody production. Establishment success rates are lower (40–60%) due to the complexity of recreating systemic autoimmune processes in vitro. Key features include elevated BAFF/APRIL signaling and defective regulatory B-cell (Breg) populations [60].

Multiple Sclerosis (MS) Models: MS organoids typically combine neural tissue organoids with immune cell infiltrates, modeling neuroinflammation and demyelination processes. These models emphasize oligodendrocyte–microglia interactions and Th1/Th17 cell infiltration across blood–brain barrier mimics [61,62].

#### 4.2.2. Patient Variability and Personalized Approaches

Immune organoids show promise in capturing heterogeneous clinical phenotypes observed in autoimmune diseases. Studies utilizing patient-derived organoids have demonstrated significant variability in drug responses that correlate with clinical treatment outcomes. For example, patient-derived organoids from seven individuals with epithelial ovarian cancer were shown to closely replicate clinical responses to platinum-based chemotherapy, supporting their predictive validity [63]. Similarly, organoids generated from patients with colorectal cancer peritoneal metastases demonstrated the capacity to forecast patient outcomes following cytoreductive surgery combined with hyperthermic intraperitoneal chemotherapy [64]. However, variability in response depends on individual clonal properties [65]. This heterogeneity underscores the potential value of organoids for personalized medicine approaches. Table 4 summarizes disease-specific organoid characteristics, success rates, and key findings.

Modeling Disease Pathogenesis has primarily involved generating organoids from affected tissues, such as the gut, pancreas, or synovium, rather than from immune tissues themselves [56]. Some co-culture studies incorporating immune cells with these organoid systems have been used to model aspects of autoimmunity, such as T-cell infiltration and cytokine dysregulation [18,43]. Mechanistic studies using immune organoids have begun to characterize autoimmune T-cell activation via hyperresponsiveness to self-antigens presented by MHC-II complexes. This involves the dysregulation of central tolerance pathways, including impaired autoimmune regulator (AIRE) expression in thymic epithelial cells and overactivation of STAT1/STAT3 in response to cytokines, such as IL-6 and IFN-γ [66,67,68]. Aberrant Th17/Treg balance, mediated through the differential activation of RORγt and FOXP3, is another hallmark that can be recapitulated in gut-associated immune organoids responding to IL-1β and IL-23 [69]. Nonetheless, the literature on pure immune organoids recreating autoimmune pathogenesis remains scarce currently.

Drug screening in autoimmune diseases using organoid models is an emerging field, though it currently has limited examples. While there is substantial research involving organoids to study disease pathogenesis and treatment responses, such as testing TNF-α inhibitors in rheumatoid arthritis models [59], applications specifically for the high-throughput drug screening of autoimmune therapies using immune organoids have not yet been widely explored. However, patient-derived organoids show significant potential for advancing personalized therapies, particularly in cancer. These models are already being employed in preclinical drug screening and for predicting patient-specific treatment responses [70,71].

Most drug screening efforts have primarily focused on tissue-derived organoids, particularly those derived from tumors [72,73], with only a few studies examining organoids derived from pluripotent stem cells [74,75]. The novelty of using patient-specific immune organoids lies in their potential to screen for sensitive drugs and offer tailored treatment options for cancer. These personalized immune organoids provide a theoretical framework for predicting therapeutic responses and identifying biomarkers. Furthermore, integrating omics technologies could enable more individualized interventions [76], though the clinical application of these approaches remains a goal for the future rather than an immediate reality.

**Table 4:** Disease-specific autoimmune organoid models.

Disease	Organoid Model Types	Key Cellular Components	Methods Used	Clinical Application	Success Rate	Drug Screening Outcomes	Therapeutic Response	Patient Variability Captured
Rheumatoid Arthritis [77,78,79]	Synovial–immune organoid	Synovial fibroblast-like synoviocytes (FLS), M1-like macrophages, endothelial cells	3D co-culture of synovial fibroblasts, endothelial cells, macrophages	Pathogenesis modeling, drug screening	High (reproducible co-cultures with FLS and immune cells)	Inflammatory readouts, including upregulated TNF-α and IL-6; relevant for drug evaluation though response rates in organoids not quantified	Recapitulates TNF-α/IL-1β-driven inflammation	Significant variability in FLS behavior across patients; RA-FLS from different individuals show heterogeneity in cytokine production and matrix remodeling
Systemic Lupus Erythematosus [57,80,81]	Germinal center-like B-cell organoid	B cells, plasma cells, dendritic cells, defective Bregs, BAFF/APRIL	Patient B cells/plasma cells + DCs in scaffold	Autoantibody study, screening B-cell-targeted therapy	In vivo variability high in BAFF expression and autoantibody profiles	Belimumab (anti-BAFF) reduces naïve/transitional B-cell fractions (~90% depletion in T3 cells), correlating with modest clinical responses (~58% achieving SRI-4 vs. ~46% placebo in trials)	BAFF-driven B-cell dysregulation; supports belimumab modeling with significant outcome	High variability in autoantibody production (10-fold difference between patients)
Multiple Sclerosis [61,62]	Neural immune assembloid	Th1/Th17 T cells, CD20^+^ B cells, activated microglia, oligodendrocytes	iPSC-derived cerebral organoid ± immune module	Pathogenesis modeling, targets for therapeutic outcome	Moderate (CNS assembloids with iPSC-derived organoids increasingly used)	Anti-CD20 therapies (e.g., rituximab, ocrelizumab) reduce B-cell activation; modeled via CD20^+^ T-cell depletion	Decrease oligodendrocyte differentiation, decrease proliferation markers, neuroinflammation modeling	Progressive vs. relapsing-remitting phenotypes show distinct organoid characteristics
Type 1 Diabetes [82,83]	Islet–immune organoid	Islet β cells, autoreactive CD8^+^/CD4^+^ T cells, Tregs	Engineered pluripotent stem cell transplant	Autoimmunity modeling, β-cell therapy screening	Moderate (islet-like organoids are established, immune integration ongoing)	Teplizumab delays T1D onset; organoid-based screening in development; β-cell destruction modeled in co-cultures	Insulin independence within 75 days post-transplantation and maintained glycemic stability for over a year	Variable degrees of insulitis among donors; age-dependent changes in immune infiltration and β-cell autoimmunity

## 5. Challenges and Limitations

While immune organoids hold immense promise as transformative platforms for studying immune responses and developing immunotherapies, their widespread adoption is hindered by several biological and technical challenges. These limitations stem from the complexity of replicating immune system functions, limited maturation of organoid models, heterogeneity among organoids, scalability challenges, and, to a lesser extent, resource demands.

### 5.1. Complexity of Immune Responses

One of the primary challenges in developing immune organoids is replicating the systemic complexity of immune interactions. The immune system operates as a dynamic, multi-layered network involving crosstalk between various cell types and organs, such as the lymph nodes, spleen, thymus, and bone marrow. Current immune organoid models often focus on localized processes, but lack the systemic integration necessary to fully recapitulate immune cell trafficking, antigen presentation across tissues, and systemic cytokine dissemination [8,26]. Moreover, interactions between the immune system and non-immune tissues, such as epithelial barriers in the gut or skin, are challenging to model without integrated multi-organoid or organ-on-a-chip approaches [84]. Additionally, many immune organoid systems are still limited by incomplete tissue complexity, missing specific immune subpopulations (e.g., stromal cells, follicular dendritic cells), or architectural features critical for faithful immune responses [85]. As a result, while immune organoids provide valuable insights, they currently model only a subset of in vivo immune functionality. Furthermore, the molecular maturation of immune cells within organoids remains incomplete. For instance, insufficient upregulation of activation markers (e.g., CD69, CD25), cytokine transcription (e.g., IL2, IFNG), and memory-associated surface proteins (e.g., CD45RO, CCR7) suggests a lack of full phenotypic and transcriptional differentiation [86]. Attempts to address this involve optimizing cytokine cocktails (e.g., IL-7, IL-15, IL-21) and ECM stiffness, which affect nuclear translocation of STATs and other transcriptional regulators [87,88,89].

### 5.2. Lack of Standardization

A significant barrier to the broader application of immune organoids is the lack of standardized protocols for their generation and functional validation. Differences in the source of cells (e.g., iPSCs, ESCs, or primary tissues), culture conditions, extracellular matrices, and differentiation protocols lead to variability in organoid structure, cellular composition, and functionality.

#### 5.2.1. Emerging Standardization Solutions

Several initiatives are addressing standardization challenges through coordinated efforts across the research community. Consensus extracellular matrix (ECM) formulations represent a major advance, with recent efforts focusing on developing standardized, defined ECM hydrogels to replace variable Matrigel preparations. Some studies have proposed guidelines for synthetic hydrogel compositions containing defined concentrations of laminin, collagen IV, and fibronectin for immune organoid culture [90,91]. These standardized matrices show 15–20% improvement in batch-to-batch reproducibility compared to undefined natural matrices.

Standardized cytokine cocktails are emerging through consensus protocols for cytokine supplementation during immune organoid differentiation. For thymic organoids, standardized combinations include BMP4 (10 ng/mL), FGF7 (25 ng/mL), and FGF10 (50 ng/mL) for epithelial development, followed by IL-7 (5 ng/mL) and SCF (100 ng/mL) for T-cell maturation [32,33]. These protocols show >80% success rates across multiple laboratories when followed precisely.

#### 5.2.2. Mitigating Donor-Specific Effects

Laboratories are implementing several strategies to reduce donor variability through systematic approaches to cell sourcing and culture standardization. iPSC standardization involves using well-characterized iPSC lines from defined genetic backgrounds to reduce donor-specific variation. Pooling multiple iPSC lines (typically 3–5 donors) in organoid cultures provides more representative population responses while maintaining experimental control [76].

For tissue-derived organoids, primary cell protocols incorporate standardized approaches, including defined tissue dissociation methods using standardized enzyme concentrations, cell sorting protocols to ensure consistent starting populations, and cryopreservation standards to maintain cell viability across experiments. Computational correction methods represent an emerging solution, with advanced analytical approaches using machine learning to identify and correct for donor-specific batch effects in organoid data, thereby improving cross-laboratory reproducibility [92,93].

Without universally accepted benchmarks, such as standardized markers for immune cell activation, antigen presentation, or cytokine production, reproducibility across laboratories remains challenging [26]. Establishing robust criteria for organoid quality control is critical for their acceptance in both academic and translational settings. A further challenge lies in the inconsistent expression of molecular markers, such as FOXP3 (regulatory T cells), T-BET (Th1 cells), and BCL6 (germinal center B cells), which can compromise functional interpretation [94,95]. Molecular phenotyping using flow cytometry or single-cell transcriptomics is essential for validation, but is not yet universally adopted in organoid workflows [96,97].

### 5.3. Scalability

Scaling up immune organoid production remains a significant barrier to their broader application in high-throughput drug screening and personalized therapeutic strategies. While manufacturing costs are an important consideration, the challenges of scalability are multifaceted, affecting the consistency, efficiency, and clinical utility of immune organoid platforms.

One critical challenge is the success rate of establishment. Creating functional immune organoids from patient-derived cells or pluripotent stem cells often yields inconsistent results. Success rates can vary widely depending on donor variability, cell quality, and the technical precision required during organoid fabrication. In some protocols, only partial efficiency is achieved, which limits the feasibility of producing immune organoids at scale for clinical or industrial use [98]. This variability underscores the need for more robust and standardized protocols that can accommodate biological heterogeneity.

Another concern is the time required for generation. The maturation of immune organoids into fully functional structures capable of simulating in vivo immune responses typically spans several weeks to months. This extended timeline poses logistical challenges for applications requiring rapid turnaround, such as patient-specific immunotherapy testing or emergency pathogen response [99]. Shortening the time to maturity without compromising organoid functionality is a key area for future development.

Heterogeneity within and between organoid batches is also a substantial limitation. Even organoids derived from the same donor or cell line can vary in size, morphology, cellular composition, and immune functionality. These inconsistencies complicate data interpretation and reduce the reliability of organoids in high-throughput or comparative studies [100]. Addressing heterogeneity through quality control measures and manufacturing standardization is crucial for increasing the translational potential of immune organoids.

A further technical limitation lies in the lack of vascularization. Most immune organoids currently lack blood vessel-like networks, which restrict nutrient and oxygen diffusion to their inner cores. This results in size constraints and the development of hypoxic regions, impeding long-term viability and functional maturation [101]. While recent efforts to introduce vascularization into organoid systems show promise, such as co-culturing immune organoids with endothelial cells, integrating vascular organoids, using co-differentiation approaches, employing microfluidic organ-on-a-chip systems, or utilizing 3D bioprinting to create perfusable channels, these methods have not yet been widely adopted or optimized for immune organoid applications [102].

However, the scalability of immune organoid systems is constrained by biological variability, time-intensive protocols, intra-batch inconsistency, and structural limitations, such as the absence of vasculature. Addressing these issues will require an interdisciplinary innovation and collaborative refinement of both the biological and engineering aspects of organoid technology.

### 5.4. Limited Complexity and Tissue Architecture

Immune organoids currently lack the full tissue complexity and organization of native immune organs. Critical structural features, such as the zonation seen in lymph nodes or the medulla–cortex organization of the thymus, are often only partially replicated [103]. Efforts are ongoing to mimic the zonation process using the oxygen biosensor device [104]. Moreover, key supporting cell types, such as fibroblastic reticular cells, endothelial cells, or specialized antigen-presenting cells, may be absent or insufficiently integrated into the organoids. These cells are crucial for adequate functioning of the immune response to organoids. This limits the fidelity of immune education, activation, and tolerance mechanisms modeled in vitro. Current efforts are aimed at improving architectural fidelity include controlling initial cell seeding density, using bioprinting approaches, and integrating stromal compartments [105], but these remain areas of active research and development.

### 5.5. Resource Demands

While the cost of materials (e.g., matrices, cytokines) and infrastructure (e.g., 3D bioreactors, advanced imaging) is non-trivial, relatively few studies have provided detailed analyses of cost as a primary barrier in immune organoid adoption. Thus, while cost may pose challenges, especially for resource-limited settings, biological and scalability challenges currently represent more significant obstacles [99].

### 5.6. Addressing the Challenges

Efforts to overcome the limitations of immune organoid development are progressing rapidly. One major barrier, the lack of vascularization, is being actively addressed through microfluidic organ-on-chip systems and perfused vascular networks, which aim to mimic physiological fluid dynamics and improve nutrient and oxygen diffusion [8,102]. Recent advances in microfluidic integration have enabled the continuous perfusion of immune–tumor co-cultures, supporting longer-term viability and more stable cytokine gradients [106]. These systems have enhanced the representation of vascularized tissue niches, yet they still fall short in the complete replication of the lymphatic drainage, which is essential for immune cell recirculation and trafficking. Studies that have attempted to do so are yet to achieve complete anatomical resemblance [107,108,109]. The absence of a functional lymphatic component may impair studies of dendritic cell migration, antigen transport, and T-cell egress, thus limiting the translational fidelity of immune activation and checkpoint response modeling.

In parallel, standardization efforts are being deployed to enhance reproducibility across laboratories. Initiatives focused on harmonizing protocols for cell sourcing, media composition, matrix selection, and functional assays are gaining momentum [89]. Such measures are critical to reducing variability and ensuring reproducible readouts, particularly in applications like drug screening and immunotherapy response prediction.

From a scalability standpoint, emerging technologies, like high-throughput 3D bioprinting, microfabrication, and robotic culture systems, are being developed to improve the uniformity and throughput of immune organoid generation [24]. Although production costs remain a barrier, collaborative efforts between academia, biotech, and regulatory agencies are fostering platforms that are more cost-efficient and industrially scalable. These advances are key to accelerating the integration of immune organoids into mainstream preclinical pipelines and, ultimately, clinical decision-making.

## 6. Future Directions

Immune organoids possess immense potential to revolutionize immunotherapy and immunological research. Fully realizing this potential will require addressing the current limitations through a systematic advancement across multiple fronts, organized into achievable timelines and addressing specific barriers to clinical adoption.

### 6.1. Timeline-Based Development Strategy

#### 6.1.1. Short-Term Goals (1–3 Years)

Priority development of standardized ECM hydrogels focuses on creating consensus extracellular matrix formulations to replace variable Matrigel preparations. A standardized concentration of ECM to fit all preparation may not be feasible as the concentration of different organoids varies and rightly depends on the organ of interest and the physical properties modeled [110].

Quality control systems for immune organoid reproducibility increasingly emphasize standardized protocols to ensure consistency across batches. Key criteria include maintaining consistent organoid size, viability, and functional capacity. For example, microwell-based fabrication methods have been employed to reduce variability in organoid morphology and ensure uniform starting cell densities, with intra-batch variation in organoid diameter often maintained below 10% to reduce necrosis and enhance functional reliability [111]. Viability thresholds of ≥70–80% post-thaw or after extended culture periods are commonly used to define batch acceptance, particularly in protocols aligned with biobanking and clinical-grade production standards [112]. These practices reflect a growing consensus toward standardizing immune organoid platforms for translational and clinical application.

The establishment of consensus cytokine protocols involves developing standardized differentiation media compositions for major organoid types, with defined concentrations, timing, and supplier specifications. Initial thymic organoid protocols have demonstrated promising levels of reproducibility under standardized laboratory conditions, with studies showing a consistent generation of thymic epithelial cell phenotypes and support for T-cell maturation in vitro. For instance, Liu et al. [113] reported a robust and uniform formation of miniaturized thymic organoids (mTOs) using a microwell-array system, suitable for medium-throughput applications. While these results affirm protocol stability within controlled settings, large-scale, multi-center validation are highly recommended.

#### 6.1.2. Medium-Term Goals (3–5 Years)

Development of perfusable organoid systems incorporating endothelial networks represents a critical advancement for vascularization integration. Current approaches, including co-culture with human umbilical vein endothelial cells (HUVECs) and bioprinting with vascular channels, show promising outcomes [102].

Integration of immune organoids with tumor, gut, and skin models will produce multi-organoid platforms that simulate systemic immune responses. Early proof-of-concept studies demonstrate successful immune cell trafficking between connected organoid compartments [84,114]. Machine learning algorithms for the real-time optimization of culture conditions represent AI-optimized production capabilities, enabling the prediction of organoid quality metrics and the identification of optimal differentiation protocols. Current systems show 25–30% improvement in success rates when compared to standard protocols [92,93,115,116].

#### 6.1.3. Long-Term Goals (5+ Years)

Complete organoid platforms with integrated immune niches, perfusion networks, and systemic circulation mimics will enable fully vascularized multi-organoid systems. These systems would facilitate the comprehensive study of immune cell trafficking, systemic cytokine responses, and organ-specific immune interactions [102,114].

Patient-derived organoid systems for routine immunotherapy prediction and personalized treatment selection represent the goal of clinical integration. A recent review asserts that the development of immune cell-integrated organoid models will enable a faithful simulation of the tumor microenvironment and integration into clinical decision-making workflows [117].

Fully automated organoid manufacturing platforms capable of producing thousands of standardized organoids per week will support industrial drug screening applications through automated production systems [118].

### 6.2. Multi-Organoid Systems

A promising avenue for advancing immune organoid research is the integration with other organ systems to create multi-organoid platforms. Such systems can more accurately model the systemic nature of immune responses, including interactions between immune cells and non-immune tissues, such as tumors, gut models, and skin. For example, coupling immune organoids with tumor-on-a-chip models enables an investigation of immune surveillance, immune evasion mechanisms, and immunotherapy responses in cancer [114]. Similarly, integrating immune organoids with gut models could elucidate the influence of the microbiome on immune function [119], offering insights into autoimmune and inflammatory diseases. Also, incorporating sphingosine-1-phosphate (S1P) signaling gradients into organoid systems may enhance the fidelity of immune cell trafficking and compartmentalization, enabling more physiologic modeling of lymphoid zonation [120]. These complex, interconnected models would also facilitate the study of systemic immune-related adverse effects, such as cytokine release syndrome, thereby improving drug safety evaluation.

### 6.3. Advanced Biomaterials

The development of advanced biomaterials tailored to support immune tissue architecture is critical for the next generation of immune organoids. The Wnt/β-catenin signaling pathway plays a critical role in maintaining progenitor cell populations and supporting the self-renewal capacity of immune organoids [121]. Fine-tuning this pathway may improve organoid yield and viability, especially during scale-up processes. Traditional matrices, such as Matrigel, while supportive, lack the biochemical and mechanical specificity required for optimal immune organoid development [90]. Future biomaterials should mimic the ECM of lymphoid tissues, supporting appropriate immune cell migration, aggregation, and activation. Innovations such as synthetic hydrogels with tunable stiffness, biofunctionalized scaffolds, and dynamic ECMs responsive to cytokine signals are particularly promising [90,122,123,124,125]. These biomaterials would enable the formation of more physiologically relevant structures, such as germinal centers, thus improving the predictive power and clinical relevance of immune organoid systems.

### 6.4. AI Integration

AI offers transformative potential for optimizing immune organoid research. AI-driven analyses can efficiently process large-scale datasets generated from immune organoid experiments, including transcriptomics, proteomics, and imaging data [115,116]. Machine learning algorithms can identify novel biomarkers, predict patient-specific responses to therapies such as checkpoint inhibitors or CAR-T-cell treatments, and guide the optimization of organoid fabrication by identifying key variables that impact reproducibility and functionality [92,93]. Furthermore, AI could assist in the real-time monitoring of organoid development and immune responses, enabling the dynamic adjustment of culture conditions to better mimic physiological states [117]. Integrating AI into the immune organoid research will thus accelerate both discovery and clinical translation.

### 6.5. Barriers to Clinical Adoption

#### 6.5.1. Regulatory Challenges

The translation of immune organoids into clinical practice faces significant regulatory hurdles that must be systematically addressed through coordinated efforts with regulatory agencies. Currently, no standardized regulatory framework exists for organoid-based diagnostic or therapeutic applications. The FDA’s 2019 guidance on “Tissue-Engineered Medical Products” provides some direction, but specific guidelines for immune organoids remain undefined. Key regulatory requirements likely include the validation of organoid-derived predictions against clinical outcomes in prospective studies, standardized manufacturing protocols meeting Good Manufacturing Practice (GMP) standards, defined quality control metrics and release criteria for clinical-grade organoids, and long-term stability and reproducibility data across multiple sites.

For organoids to be used as predictive biomarkers, they must meet companion diagnostic requirements, including analytical validity, clinical validity, and clinical utility. This process typically requires 3–5 years and substantial investment (running into millions of USD) for regulatory approval [126].

Coordination between the FDA, EMA, and other international regulatory bodies will be essential for global adoption through international harmonization efforts. The International Council for Harmonisation (ICH) guidelines may need updating to address organoid-specific considerations.

#### 6.5.2. Economic Considerations

While precise cost estimates vary between centers, it is understood that establishing patient-derived organoid lines for drug sensitivity assays entails substantial initial expenses due to specialized infrastructure and reagents. Importantly, if organoid testing helps prevent high-cost treatment failures, particularly in immunotherapy, where expenditures can exceed six figures per patient, the approach may become economically justifiable over a defined patient cohort. Efforts toward automation and scale-up are projected to reduce per-organoid costs and improve consistency, although quantifiable capital and operational metrics are not yet publicly documented. Ultimately, integration into standard care pathways will hinge on the outcomes of prospective clinical validation studies and health technology assessments demonstrating both clinical utility and cost-effectiveness.

#### 6.5.3. Ongoing Clinical Validation Studies

Several clinical studies are actively evaluating the translational potential of organoid technology, particularly in oncology, though the clinical validation of immune organoids remains limited.

One notable effort is the SENSOR Trial (Single-Patient Experimental Therapy Using Organoids in Refractory Cancer), a prospective pilot study in the Netherlands investigating the feasibility of using patient-derived tumor organoids to inform drug selection in metastatic colorectal cancer patients [127]. Among 54 eligible patients, organoids were successfully generated in 31 cases, 25 cultures underwent drug screening, and 19 exhibited in vitro responses to at least one compound. However, six patients treated based on these results did not achieve objective clinical responses, highlighting the current limitations of organoid-guided therapy in complex metastatic diseases.

In Europe, coordinated efforts, such as the Human Cancer Models Initiative (HCMI), and similar consortium-based programs aim to standardize tumor organoid generation and implement a large-scale, multicenter validation of organoid-based drug sensitivity testing [128]. These initiatives focus primarily on solid tumors, with growing interest in expanding toward immunotherapy applications, although trials specifically targeting immune organoid models remain at an early stage.

In the United States, organoid research is supported by various NIH programs, including the NIH 4D Nucleome, Cancer Moonshot, and Common Fund initiatives. Multiple NIH-funded consortia support the development of organoid models for disease modeling, therapeutic screening, and regulatory readiness, including over two dozen projects listed in public NIH portfolios [129].

Together, these programs underscore the translational promise of organoid platforms while also emphasizing the need for larger, well-controlled clinical studies to establish predictive accuracy and reproducibility across disease contexts, including immune-mediated conditions.

### 6.6. Clinical Translation

Achieving clinical translation of immune organoids requires overcoming the current limitations in their scalability, standardization, and reproducibility. While preclinical studies have shown their potential, particularly in personalized immunotherapy and disease modeling, they need to be translated into practical clinical tools and validated through clinical trials. For example, patient-derived immune organoids could be used to test responses to immunotherapies ex vivo, predicting which patients are most likely to benefit from specific treatments [130]. Large-scale validation studies, along with the establishment of robust biobanking practices [131,132], will be necessary to integrate organoid technologies into clinical workflows. Moreover, regulatory agencies are increasingly interested in 3D models for drug development [126], but standardized manufacturing practices and reproducibility benchmarks must be achieved to gain widespread regulatory acceptance.

### 6.7. Long-Term Vision

The long-term vision for immune organoids is their incorporation into personalized medicine frameworks, where patient-specific organoid models inform real-time therapeutic decisions. Multi-organoid systems simulating interactions between different tissues, advanced biomaterials mimicking native tissue ECMs, AI-driven optimization, and rigorous clinical validation will collectively enable the widespread clinical use of immune organoids. Such developments would revolutionize precision immunotherapy, enhance our understanding of immune aging, improve vaccine development pipelines, and offer new models for studying emerging infectious diseases. For example, the modeling of host–pathogen interactions can be improved by engineering immune organoids to express toll-like receptors (TLRs), facilitating innate immune activation in response to microbial ligands or synthetic adjuvants [133]. As interdisciplinary collaboration across bioengineering, immunology, and computational biology continues to evolve, immune organoids are poised to become indispensable tools in next-generation healthcare. Future work may also explore epigenetic regulation, including chromatin accessibility and histone modifications, to more accurately reproduce lineage commitment and immune memory formation in organoid-based models.

Table 5 summarizes key signaling pathways relevant to immune organoid development and potential applications, now expanded to include therapeutic targeting potential.

## 7. Limitations of the Review

This narrative review provides a broad and integrative overview of immune organoids and their applications in cancer immunotherapy and autoimmune disease research. However, several limitations should be acknowledged. First, the review is not systematic and therefore lacks the methodological rigor of a formal systematic review or meta-analysis. The selection of studies was based on thematic relevance and expert judgment, which may introduce selection bias and limit reproducibility. Although efforts were made to include high-impact and peer-reviewed literature, the absence of a formal quality assessment tool means that variability in the methodological quality of included studies could influence the conclusions drawn.

Second, the rapidly evolving nature of organoid research poses a challenge to capturing the most recent advancements. Some studies, especially those in preprint or early publication stages, may not have been included despite offering valuable insights. Furthermore, given the diversity of immune organoid models, applications, and fabrication methods, this review could not exhaustively cover all technological variations or disease-specific implementations.

Third, while this review draws connections between immune organoid systems and their translational potential, it does not include a quantitative evaluation of outcomes, such as effect sizes or comparative efficacy, due to the narrative approach. This limits its utility for guiding clinical decision-making or for comparing different organoid models on a standardized scale.

Finally, this review focuses predominantly on human models and does not account for developments in animal-derived organoid systems, which may also contribute valuable insights, particularly in early-stage immunological research. These limitations underscore the need for complementary systematic reviews and experimental studies to build on the themes discussed and validate the translational promise of immune organoids.

## 8. Conclusions

Immune organoids represent a ground-breaking advancement in immunology, offering high-fidelity, physiologically relevant models to study human immune responses. By bridging the critical gaps left by traditional two-dimensional culture and animal models, immune organoids enable the detailed investigation of immune mechanisms, disease pathogenesis, and therapeutic interventions. Their applications in cancer immunotherapy, autoimmune disease research, and infectious disease modeling highlight their transformative potential, allowing researchers to develop, optimize, and personalize treatments with unprecedented precision. While challenges related to complexity, scalability, heterogeneity, standardization, and biological maturation remain, ongoing advancements in bioengineering, biomaterials, AI, and clinical integration are steadily addressing these limitations through systematic, timeline-based approaches.

The integration of immune organoids into multi-organ systems, coupled with emerging solutions for standardization and scalability challenges, positions these platforms for clinical translation within the next decade. Regulatory frameworks are evolving to accommodate organoid-based diagnostics, while economic analyses support their cost-effectiveness for personalized medicine applications. As the field progresses, immune organoids are poised not only to revolutionize immunotherapy and autoimmune disease management, but also to reshape broader areas of biomedical research and clinical care, including regenerative medicine and vaccine development. Ultimately, these innovative systems hold the promise of improving patient outcomes and ushering in a new era of personalized, effective medical care, with concrete pathways toward clinical implementation now clearly defined.

## Figures and Tables

**Table 1:** Immune organoids vs. traditional models.

Feature	Immune Organoids	Traditional 2D Cultures	Animal Models
Cellular Interaction [8]	Supports complex, dynamic, and spatially organized interactions among diverse immune cells.	Limited interaction; cells grown in monolayers lack 3D spatial organization.	Includes systemic immune interactions but differs from human physiology, limiting translational relevance.
Physiological Relevance [22]	Closely mimics human immune microenvironments, enabling personalized research.	Poor mimicry of in vivo systems; lacks tissue architecture.	Limited due to interspecies differences in immune system components and responses. Varies based on system, animal and pathology being investigated.
Scalability [8,23,24]	Moderate; advances in bioprinting and automation are improving scalability.	High; simple setup allows large-scale experiments.	Low; requires significant resources for maintenance and ethical considerations.
Predictive Value for Humans [25]	High; accurately models human immune responses and therapeutic outcomes.	Low; fails to capture immune complexity.	Moderate; often fails to predict human-specific outcomes, particularly for immunotherapies.

**Table 2:** Types of immune organoids and their applications.

Type of Immune Organoid	Source Material	Applications	Key Example Studies
Lymphoid Organoids [26,36]	iPSCs, lymph node tissues, or spleen-derived cells	Studying lymphocyte development, antigen presentation, and adaptive immune responses	Germinal center formation observed in lymphoid organoids derived from tonsil tissue
Thymic Organoids [39]	iPSCs or ESCs	Modeling T-cell maturation and thymic involution during aging or disease	Thymic organoids supporting T-cell differentiation from hematopoietic progenitor cells
Tumor–Immune Organoids [40,41]	Co-culture of tumor-derived and immune cells	Testing immunotherapies like CAR-T cells and checkpoint inhibitors in tumor-like immune environments	Tumor–immune organoids used to evaluate PD-1 inhibitors and CAR-T cell cytotoxicity
Gut-Associated Immune Organoids [42]	iPSCs or primary intestinal and immune cells	Modeling gut–immune interactions, such as microbiome effects on immune responses and inflammatory bowel disease research	Gut–immune organoids used to study cytokine profiles in response to inflammatory stimuli
Autoimmune Disease Organoids [43]	Patient-derived immune cells or tissues	Exploring immune dysregulation and screening immunosuppressive therapies for autoimmune diseases like lupus or rheumatoid arthritis	Autoimmune organoids mimicking cytokine storm phenomena in rheumatoid arthritis models

**Table 3:** Clinical readiness of immune organoid models for cancer immunotherapy applications.

Immunotherapy Strategy	Application in Immune Organoids	Clinical Readiness
Checkpoint Inhibitors [48]	T-cell activation; PD-1/PD-L1 blockade	Exploratory—limited retrospective correlation
CAR-T-Cell Therapy [49,50,51]	Antigen-specific activation; cytotoxicity profiling	Exploratory—no clinical correlation yet
Cancer Vaccines [56,57]	Antigen presentation; T-cell priming	Early stage—conceptual exploration only

**Table 5:** Key signaling pathways in immune organoid development, function, and therapeutic targeting potential.

Pathway	Role in Immune Organoids	Potential Applications	Therapeutic Targeting Potential
Wnt/β-Catenin [89,134]	Maintains stem/progenitor cell renewal and supports tissue regeneration	Enhancing proliferation, longevity, and structural self-organization of organoids	Wnt modulators (e.g., LGK-974) could optimize organoid establishment and maintenance in aging-related immunodeficiency models
Notch [135]	Regulates T-cell lineage commitment, thymic epithelial development, and splenic marginal B-cell development	Thymic organoid models, T-cell education, and lymphoid structure formation	Notch inhibitors (e.g., DAPT, DBZ) may improve thymic organoid function in aging models by enhancing T-cell maturation efficiency
Integrin–ECM Signaling [136]	Mediates cell adhesion, migration, and spatial patterning via interactions with ECM	Structural maturation, immune cell compartmentalization, and signal transduction	Integrin antagonists could be screened in organoids for anti-inflammatory effects in autoimmune disease models
Sphingosine-1-Phosphate [120]	Controls immune cell trafficking and compartmentalization	Modeling lymphoid zonation, immune cell egress, and germinal center dynamics	S1P receptor modulators (e.g., fingolimod analogs) can be tested for efficacy in organoid models of multiple sclerosis and transplant rejection
Toll-Like Receptors (TLRs) [133]	Activate innate immune responses to microbial or damage-associated ligands	Modeling infection, inflammation, and immune adjuvant responses	TLR agonists and antagonists can be screened in organoids for vaccine adjuvant development and sepsis therapeutics
BAFF/APRIL [81,137]	Support B-cell survival, maturation, and antibody class switching	Simulating germinal center activity; antibody production in lymphoid organoids	BAFF/APRIL inhibitors (e.g., belimumab, atacicept) can be evaluated in lupus organoid models for personalized therapy selection
IL-7/IL-2/IL-21 Gradients [88]	Regulate T- and B-cell activation, differentiation, and homeostasis	Creating immune niches, improving germinal center formation, and supporting functional immune responses	Cytokine receptor agonists and antagonists can be tested for immunodeficiency treatments and cancer immunotherapy optimization
PI3K–AKT/MAPK [138]	Downstream of integrin and cytokine signaling; promote survival, proliferation, and motility	Enhancing cellular viability, migration, and response to external stimuli in immune organoids	PI3K/mTOR inhibitors can be screened in organoid models for cancer immunotherapy combinations and autoimmune disease treatments
Epigenetic Regulators (e.g., HDACs, DNMTs) [139,140]	Govern gene accessibility and immune lineage commitment	Improving fidelity of immune differentiation, modeling memory formation. or tolerance induction	Epigenetic modulators (e.g., HDAC inhibitors, DNA methyltransferase inhibitors) can be evaluated for immune system rejuvenation and memory enhancement therapies
